# Compositional movement behaviours and preschool children’s social-emotional development

**DOI:** 10.1186/s12966-026-01911-2

**Published:** 2026-04-16

**Authors:** Hayley Christian, Emma K. Adams, Stephen Vander Hoorn, Phoebe George, Andrea Nathan, Stewart G. Trost, Jasper Schipperijn, Valerie Carson

**Affiliations:** 1 The Kids Research Institute Australia, The University of Western Australia, Perth, WA Australia; 2 School of Population and Global Health, The University of Western Australia, Perth, WA Australia; 3 School of Human Movement and Nutrition Sciences, University of Queensland, Brisbane, QLD Australia; 4 Department of Sports Science and Clinical Biomechanics, University of Southern Denmark, Odense, Denmark; 5 Faculty of Kinesiology, Sport, and Recreation, University of Alberta, Edmonton, AB Canada

**Keywords:** Compositional data analysis, Preschool children, Physical activity, Sedentary time, Sleep, Movement behaviour, Development

## Abstract

**Background:**

Individually, sufficient physical activity and sleep and limited sedentary behaviour are associated with favourable social-emotional development in preschool-aged children. However, these behaviours are interdependent. Thus, we examined the effect of preschool children’s 24-hour movement behaviour compositions (physical activity, sedentary time, sleep) on social-emotional development.

**Methods:**

Wave one data from the PLAY Spaces and Environments for Children’s Physical Activity (PLAYCE) cohort study (Perth, Western Australia) were utilised (1,073 children aged 2-5-years). Daily physical activity, sedentary time, and sleep were measured using accelerometry and parent-report surveys. Social-emotional development was measured using the parent-reported Strengths and Difficulties Questionnaire. Compositional data analysis examined associations between movement behaviour compositions and social-emotional development.

**Results:**

Movement behaviour compositions were significantly associated with total difficulties (*p* = 0.008), emotional problems (*p* = 0.04), peer problems (*p* = 0.031) and hyperactivity (*p* = 0.002). More sleep and energetic play, relative to other movement behaviours, were associated with better social-emotional development. Reallocating light-intensity activity and games to sleep and sedentary behaviour were associated with better social-emotional development. After separating sedentary behaviour into screen time and quiet play, reallocating screen time and light-intensity activity and games in favour of quiet play were associated with better social-emotional development.

**Conclusion:**

Reallocating some of the daily time spent in light-intensity activities and games and screen time to sleep and energetic play could be beneficial for preschool children’s social-emotional development. Future movement behaviour composition studies should examine different types of sedentary behaviour as well as the longitudinal impact of movement behaviour composition on children’s social-emotional development.

**Supplementary Information:**

The online version contains supplementary material available at 10.1186/s12966-026-01911-2.

## Background

The 24-hour Movement Guidelines for the Early Years consider sleep, sedentary behaviour, and physical activity as interdependent behaviours [1–4]. These guidelines prioritise meeting all three movement behaviour recommendations for optimal child health and development [5]. Throughout a 24-hour period, children continuously engage in movement behaviours of different intensities, from sleeping through to vigorous-intensity physical activity. A change in the amount of time spent in one behaviour necessarily requires a change in another behaviour. Using compositional data analysis (CoDA) it is possible to examine the combination of movement behaviours across the whole day, and which substitutions in movement behaviours benefit health and development. Two recent systematic reviews identified that young children were underrepresented in studies using CoDA to examine associations between movement behaviours and health outcomes [6, 7]. Among studies with preschool-aged children, measured outcomes included body composition [8–13], fundamental movement skills [14–16], physical fitness [12, 17], and executive function [18]. Overall these studies found reallocating time from other movement behaviours (sedentary behaviour, light intensity physical activity and sleep) to moderate-to-vigorous intensity physical activity was positively associated with preschool children’s health and development.

Other development-based outcomes, including social-emotional development, have been relatively unexplored in studies using CoDA. Of the three studies conducted in preschool-aged children to date, two studies reported that movement behaviour compositions explained limited variation in social-emotional development [19, 20], while the other showed that when attending childcare, more sedentary time relative to other movement behaviours was associated with improved psychosocial functioning [21]. These mixed findings may be due in part to methodological limitations, including small [20] and population-specific (e.g., Developmental Coordination Disorder risk [19]) samples, the inclusion of wake-time movement behaviours only [19, 21] and movement behaviour data collected in specific behaviour settings (e.g., childcare centre) [21]. While a strength of these studies is the use of device-based movement behaviour data, such data cannot capture different types of sedentary time. For example, sedentary time can encompass both screen-based (e.g., tablet, phone) and non-screen-based behaviours such as drawing, reading and storytelling, with the latter beneficial for children’s social-emotional development [22]. Combining both device-based and subjective measures of sedentary behaviour (e.g., screen-based and non-screen based) could help explain if and how sedentary behaviours relative to other movement behaviours impacts preschool children’s social-emotional development. Thus, the aims of this study were to investigate: 1) the associations between preschool children’s movement behaviour compositions and social-emotional development using accelerometer data and parent-report surveys and 2) the role of different types of sedentary behaviours on these associations.

## Methods

### Study design and sample

Wave 1 (baseline) data from the PLAYCE cohort study (Perth, Western Australia) were utilised. The PLAYCE cohort study investigated young children’s movement behaviours across behaviour settings and the relationships with health and development over time. Recruitment occurred through early childhood education and care (ECEC) services using a representative sample of services based on size and socio-economic status [23]. Wave 1 data were collected 2015–2018 for 1,918 preschool children aged 2–5 years old. Further details about the PLAYCE cohort protocol have been published previously [23, 24]. 

### Measures

#### Social-emotional development

Parents reported their child’s social-emotional development using the Strengths and Difficulties Questionnaire (SDQ) [25]. The SDQ is an extensively used and validated measure of children’s social and emotional wellbeing [26]. The SDQ contains 25 items measuring five subscales: emotional symptoms, conduct problems, hyperactivity-inattention, peer relationship problems and prosocial behaviour. A total difficulties score is also derived from the sum of all sub-scales except prosocial behaviour [25]. Higher scores (lower for the prosocial behaviour subscale) correspond with higher levels of social-emotional difficulties and lower scores (higher for prosocial behaviour) correspond with lower levels of social-emotional difficulties [25]. 

#### Movement behaviours

Preschool children’s movement behaviour was measured using ActiGraph GT3X+ accelerometers (ActiGraph Corporation, Pensacola, FL USA) fitted to the right hip. Children wore the device during waking hours for seven days except for water-based activities.

Accelerometer data historically have been processed using counts per minute and classified into activity intensities via cut-points (e.g., sedentary, light, moderate, and vigorous physical activity) [27]. However, counts-based and cut-point approaches are device-specific, lack standardisation, and show limited accuracy in characterising the intensity and type of physical activity in young children, including overestimation of moderate-to-vigorous activity and underestimation of light-intensity activity [28–30]. To address these limitations, raw tri-axial acceleration signals (30 Hz) were processed using a validated random forest physical activity classification model specifically trained for activity recognition in children under five [28, 29]. Details of the application of the validated random forest physical activity classification model for processing accelerometer data from the PLAYCE cohort study have been published [31]. The model classifies five movement behaviours: sedentary (lying or sitting down); light-intensity activities and games (e.g., standing, slow walking, standing arts and crafts); walking; running; and moderate-to-vigorous activities and games (e.g., active games) [28, 29]. Energetic play was calculated by summing daily time spent walking, running and moderate-to-vigorous activities and games. Non-wear time was calculated by summing the time periods in which the standard deviation of the accelerometer signal was < 13 mg for ≥ 30 consecutive minutes [32]. Children who had a minimum of three weekdays and at least one weekend day with accelerometer wear time of eight hours or more were included [23, 24]. In a free-living evaluation, the random forest classifier had an overall classification accuracy exceeding 80% and was more precise at classifying physical activity intensity compared to existing cut-point methods [29]. 

Parents reported time their child usually slept in a 24-hour period during the night and day using established items [23, 33]. Parents also reported their child’s usual weekly amount of sedentary screen time (i.e., TV/DVD, game consoles, computers, tablets, smartphones) and other sedentary non-screen time (i.e., quiet play with books, board games, art/craft, etc.) [34]. 

#### Covariates

Parents reported their child’s age and sex and their own highest level of education (secondary school, certificate level, tertiary degree).

### Analysis

A total of 1,073 children with complete data (SDQ, valid accelerometer, parent-report sleep, child age and sex, parent education) were included. CoDA was used to analyse daily time spent in each movement behaviour. Time spent in movement behaviours was normalised to the proportion of the total day (1440 min). Non-wear time was proportionally reallocated to day-time movement behaviours (sedentary, light-intensity activities and games, energetic play) as described and recommended by Haszard et al. [35] Isometric log-ratios (ilr) were derived for each child to express the proportion of time spent in one behaviour relative to other behaviours.

Separate compositional linear regression models (four in total) with each movement behaviour as the dominant behaviour were used to examine the relationship with SDQ total difficulties and subscale scores. The R-squared and overall significance were initially estimated for the linear regression models without covariates. Each model then controlled for child age and sex, and parent education. The coefficient of the first pivot coordinate (ilr) was examined to determine if time spent in each movement behaviour relative to the remaining movement behaviours was significantly associated with the outcome.

Compositional isotemporal substitution models [36] (six in total) were run to estimate if the changes to SDQ scores associated with increasing time in one movement behaviour were contingent on which movement behaviour it replaced. The estimated changes with reallocations between movement behaviours and their 95% confidence intervals were calculated using methods described by Dumuid and colleagues [37]. Ten-minute and up to 30-minute time reallocations were presented in an attempt to account for the large difference in the mean daily time spent in different movement behaviours. The effect of replacing one movement behaviour with another movement behaviour was considered significant when the 95% confidence interval did not include zero. Analyses were carried out in R using codaredistlm [38] and compositions [39] packages. The statistical analysis and presentation are consistent with the CHAMP statement [40]. 

#### Secondary analysis

Since accelerometers do not provide information on the type of sedentary behaviour, a secondary analysis was carried out to disaggregate sedentary behaviour into a proxy for screen-based and non-screen-based sedentary behaviour (i.e., quiet play) using parent-report survey data. Usual time spent using screens (including television, game consoles, computers, tablets, and smartphones, and excluding movement-based game consoles) and in quiet play (e.g., reading, craft, sitting-based games) were proportionally applied to the device-measured sedentary time to obtain proxy measures for screen-based and quiet play sedentary time. Ten children had no parent-report sedentary time data and were excluded. Zero values cannot be included in log ratios [41]. Thus, given less than 5% of data were reported as zero, zero minutes of screen time (*n* = 10, 0.9%) or quiet play (*n* = 41, 3.9%) were replaced with a small value of 1 min. The regression and isotemporal substitution analyses were repeated using compositions of sleep, screen time, quiet play, light-intensity activities and games, and energetic play (*n* = 1,063).

## Results

Overall, 51.1% of the sample were boys and 48.9% were girls. The median age of children was 3.3 years (IQR = 1.1). Most parents had a tertiary or higher education (58.2%), 28.0% had a certificate level, and 13.8% had a secondary school level education. Unadjusted means for sleep, sedentary behaviour, light-intensity activities and games and energetic play were 687.9 (SD = 75.5), 291.3 (SD = 62.4), 334.2 (SD = 46.6) and 39.4 (SD = 14.2) minutes/day, respectively. After reallocating non-wear to day-time components, geometric means for sleep, sedentary behaviour, light-intensity activities and games, and energetic play were 692.3, 326.3, 379.0 and 42.4 min, respectively. Children spent on average 48% of the day sleeping, 23% sedentary, 26% in light-intensity activities and games and 3% in energetic play. The total difficulties unadjusted mean score was 8.7 (standard deviation, SD = 4.5). For subscale scores, the unadjusted means were 1.4 for emotional problems (SD = 1.4), 1.3 for peer problems (SD = 1.4), 2.1 for conduct problems (SD = 1.8), 3.9 for hyperactivity (Sd = 2.2), and 7.4 for prosocial behaviour (SD = 1.8).

### Associations between movement behaviour compositions and social-emotional development

Preschool children’s movement behaviour compositions were significantly associated with the SDQ total difficulties score (r2 = 0.011, *p* = 0.008), emotional problems (r2 = 0.008, *p* = 0.040), peer problems (r2 = 0.008, *p* = 0.031) and hyperactivity (r2 = 0.014, *p* = 0.002) subscale scores (Table 1). Spending a greater proportion of time asleep relative to other movement behaviours was associated with lower (better) total difficulties, hyperactivity, and peer problems scores. Spending a greater amount of time in light-intensity activities and games relative to other movement behaviours was associated with higher (worse) total difficulties and hyperactivity scores and spending more time sedentary relative to other movement behaviours was associated with worse peer problems scores. Spending more time in energetic play relative to other movement behaviours was associated with better emotional problems scores.

**Table 1:** Overall model fit results from the compositional regression analysis for each SDQ scale score

Outcome	*P*-value^1^	R2^1^	R2c^2^	Ilr1 (SLP)^3^	Ilr1 (SB)^3^	Ilr1 (LIAG)^3^	Ilr1 (ENER)^3^
Total difficulties	**0**.**008**	0.011	0.039	**-2.65 (-4.26**,** -1.05)**	0.38 (-0.87, 1.62)	**2.88 (1.29**,** 4.47)**	-0.61 (-1.52, 0.31)
Emotional problems	**0**.**040**	0.008	0.016	-0.16 (-0.66, 0.34)	0.04 (-0.35, 0.43)	0.47 (-0.03, 0.96)	**-0.35 (-0.63**,** -0.06)**
Peer problems	**0**.**031**	0.008	0.034	**-0.71 (-1.21**,** -0.22)**	**0.53 (0.15**,** 0.92)**	0.39 (-0.10, 0.87)	-0.20 (-0.48, 0.08)
Conduct problems	0.106	0.006	0.021	-0.70 (-1.34, -0.06)	0.19 (-0.30, 0.69)	0.46 (-0.17, 1.08)	0.05 (-0.31, 0.42)
Hyperactivity	**0**.**002**	0.014	0.060	**-1.08 (-1.87**,** -0.28)**	-0.39 (-1.01, 0.23)	**1.58 (0.79**,** 2.36)**	-0.11 (-0.56, 0.34)
Prosocial behaviour	0.570	0.002	0.047	0.58 (-0.06, 1.21)	-0.49 (-0.98, 0.00)	-0.05 (-0.68, 0.57)	-0.03 (-0.40, 0.33)

Significant associations (*p* < 0.05) are bolded

Models adjusted for child age and sex, and parent education

Lower scores are better for all scores except prosocial behaviour where a higher score is better

*SLP *Sleep, *SB *Sedentary behaviour, *LIAG *light-intensity activities and games, *ENER *energetic play

^1^ From model without covariates

^2^ From model with compositional variables and covariates

^3^ Isometric log-ratio (ilr) for first pivot coordinate from model with compositional variables and covariates

### One-to-one reallocations of movement behaviour compositions and the relationship with social-emotional development

**Fig. 1:**
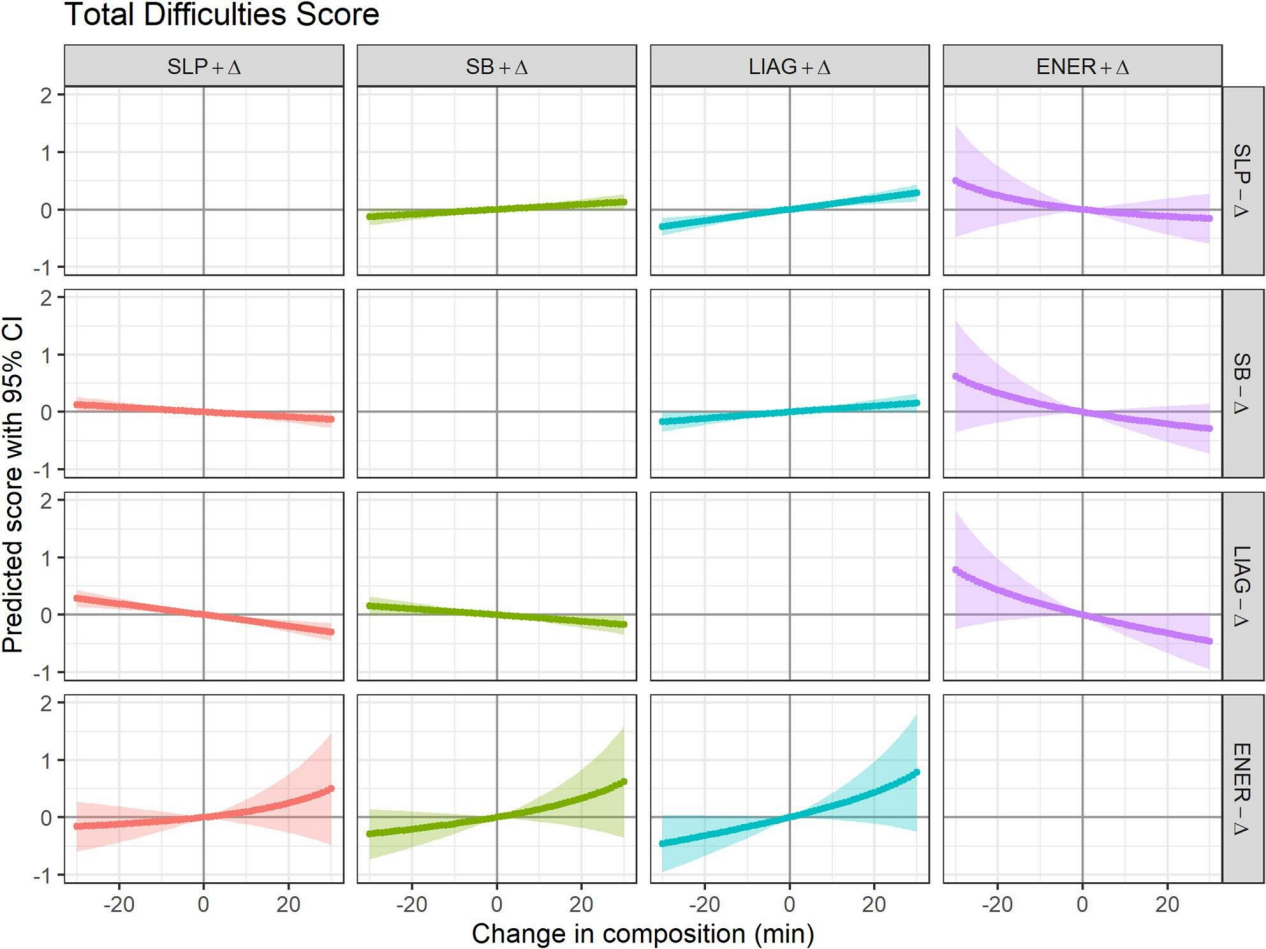
Effects of one-to-one reallocations of time between movement behaviours on SDQ Total Difficulties score; Δ = change. SLP=Sleep, SB=Sedentary behaviour, LIAG=light-intensity activities and games, ENER=energetic play

Reallocating 10 min to sleep from sedentary behaviour improved peer problems (mean change (Δ)=-0.02; 95% CI -0.04, -0.01) and prosocial behaviour (Δ = 0.02; 95% CI 0.00, 0.04) scores (Table 2). Reallocating 10 min to sleep from light-intensity activities and games improved total difficulties (Δ=-0.10; 95% CI -0.15, -0.05), peer problems (Δ=-0.02; 95% CI -0.03, 0.00), and hyperactivity problems (Δ=-0.05; 95% CI -0.07, -0.03) scores. Reallocating 10 min from any of the other movement behaviours to energetic play improved emotional problems scores by 0.06 to 0.07 points with positive trends for improved total difficulties and peer problems scores with increasing time reallocated. Reallocating 10 min of light-intensity activities and games to sedentary time improved total difficulties (Δ=-0.06; 95% CI -0.11, 0.00) and hyperactivity problems (Δ=-0.05; 95% CI -0.07, -0.02). Results for up to 30 min of time reallocated between movement behaviours are in Fig. 1 for the total difficulties score only (SDQ subscales - Additional File 1).

**Table 2:** Estimated changes (95% CI) in SDQ scores associated with 10-minute one-to-one reallocations between movement behaviours

10-minute reallocation	Total difficulties	Emotional problems	Peer problems	Conduct problems	Hyperactivity	Prosocial behaviour
SB to SLP	-0.04 (-0.09, 0.00)	0.00 (-0.02, 0.01)	**-0.02 (-0.04**,** -0.01)**	-0.01 (-0.03, 0.00)	0.00 (-0.03, 0.02)	**0.02 (0.00**,** 0.04)**
LIAG to SLP	**-0.10 (-0.15**,** -0.05)**	-0.01 (-0.03, 0.00)	**-0.02 (-0.03**,** 0.00)**	-0.02 (-0.04, 0.00)	**-0.05 (-0.07**,** -0.03)**	0.01 (-0.01, 0.03)
ENER to SLP	0.11 (-0.11, 0.32)	**0.08 (0.01**,** 0.15)**	0.04 (-0.03, 0.10)	-0.02 (-0.11, 0.06)	0.01 (-0.09, 0.12)	0.02 (-0.07, 0.10)
SLP to SB	0.04 (0.00, 0.09)	0.00 (-0.01, 0.02)	**0.02 (0.01**,** 0.04)**	0.01 (0.00, 0.03)	0.00 (-0.02, 0.03)	**-0.02 (-0.04**,** 0.00)**
LIAG to SB	**-0.06 (-0.11**,** 0.00)**	-0.01 (-0.03, 0.01)	0.00 (-0.01, 0.02)	-0.01 (-0.03, 0.02)	**-0.05 (-0.07**,** -0.02)**	-0.01 (-0.03, 0.01)
ENER to SB	0.15 (-0.06, 0.36)	**0.08 (0.01**,** 0.15)**	0.06 (0.00, 0.13)	-0.01 (-0.09, 0.08)	0.02 (-0.09, 0.12)	0.00 (-0.09, 0.08)
SLP to LIAG	**0.10 (0.05**,** 0.15)**	0.01 (0.00, 0.03)	**0.02 (0.00**,** 0.03)**	0.02 (0.00, 0.04)	**0.05 (0.02**,** 0.07)**	-0.01 (-0.03, 0.01)
SB to LIAG	0.05 (0.00, 0.11)	0.01 (-0.01, 0.03)	-0.01 (-0.02, 0.01)	0.01 (-0.02, 0.03)	**0.05 (0.02**,** 0.07)**	0.01 (-0.01, 0.03)
ENER to LIAG	0.21 (-0.03, 0.44)	**0.09 (0.02**,** 0.16)**	0.06 (-0.02, 0.13)	0.00 (-0.09, 0.09)	0.06 (-0.05, 0.18)	0.01 (-0.09, 0.10)
SLP to ENER	-0.08 (-0.25, 0.09)	**-0.06 (-0.12**,** -0.01)**	-0.03 (-0.08, 0.02)	0.02 (-0.05, 0.09)	-0.01 (-0.09, 0.08)	-0.01 (-0.08, 0.05)
SB to ENER	-0.12 (-0.29, 0.05)	**-0.06 (-0.12**,** -0.01)**	-0.05 (-0.10, 0.00)	0.00 (-0.06, 0.07)	-0.01 (-0.09, 0.07)	0.01 (-0.06, 0.07)
LIAG to ENER	-0.18 (-0.37, 0.01)	**-0.07 (-0.13**,** -0.02)**	-0.05 (-0.10, 0.01)	0.00 (-0.08, 0.07)	-0.06 (-0.15, 0.04)	0.00 (-0.08, 0.07)

Significant associations (*p* < 0.05) are bolded

Models adjusted for child age and sex, and parent education

Lower scores are better for all scores except prosocial behaviour where a higher score is better

*SLP *Sleep, *SB *Sedentary behaviour, *LIAG *light-intensity activities and games, *ENER *energetic play

### Secondary analysis

Average daily sedentary time was disaggregated into 144.5 min of screen-based sedentary time (SD = 71.0) and 147.0 min of quiet play (SD = 68.7; geometric means 141.3 and 137.0 min, respectively). Spending greater amounts of time in quiet play relative to other movement behaviours was associated with better total difficulties, conduct problems, and hyperactivity scores (Table 3). Screen time relative to other movement behaviours was not associated with social-emotional development. All other significant associations were the same as those in the main results.

**Table 3:** Overall model fit results from the compositional regression analysis after disaggregating sedentary time into screen and quiet play sedentary time

Outcome	*P*-value1	R21	R2c2	Ilr1 (SLP)3	Ilr1 (SCR)3, 4	Ilr1 (QP)3, 4	Ilr1 (LIAG)3	Ilr1 (ENER)3
Total difficulties	**0.002**	0.014	0.040	-1.13 (-2.30, 0.05)	0.20 (-0.17, 0.57)	**-0.51 (-0.80, -0.23)**	**3.05 (1.54, 4.55)**	0.30 (-0.48, 1.08)
Emotional problems	0.169	0.005	0.013	0.13 (-0.24, 0.50)	0.05 (-0.07, 0.17)	-0.06 (-0.15, 0.03)	0.48 (0.01, 0.95)	-0.20 (-0.44, 0.05)
Peer problems	0.246	0.004	0.027	-0.18 (-0.54, 0.19)	0.11 (0.00, 0.23)	-0.03 (-0.12, 0.06)	0.51 (0.05, 0.98)	-0.07 (-0.31, 0.16)
Conduct problems	**0.001**	0.015	0.028	-0.33 (-0.79, 0.14)	0.04 (-0.11, 0.19)	**-0.18 (-0.29, -0.07)**	0.57 (-0.03, 1.16)	0.23 (-0.08, 0.53)
Hyperactivity	**0.008**	0.011	0.057	-0.75 (-1.34, -0.17)	0.00 (-0.18, 0.19)	**-0.24 (-0.38, -0.10)**	**1.48 (0.74, 2.23)**	0.34 (-0.04, 0.73)
Prosocial behaviour	0.545	0.002	0.047	0.31 (-0.15, 0.78)	-0.13 (-0.28, 0.01)	-0.02 (-0.14, 0.09)	-0.14 (-0.73, 0.46)	-0.04 (-0.35, 0.26)

Significant associations (*p* < 0.05) are bolded

Models adjusted for child age and sex and parent education

Lower scores are better for all scores except prosocial behaviour where a higher score is better

*SLP *Sleep, *SCR *screen-based sedentary behaviour, *QP *quiet play-based sedentary behaviour, *LIAG *light-intensity activities and games, *ENER *energetic play

^1^ From model without covariates

^2^ From model with compositional variables and covariates

^3^ Isometric log-ratio (ilr) for first pivot coordinate

4 Zero minutes of screen time or quiet play were replaced with a value of 1 min

Reallocating 10 min towards quiet play away from screen time or light-intensity activities and games was associated with better total difficulties (Δ=-0.04; 95% CI -0.07, -0.02; Δ=-0.09; 95% CI -0.13, -0.05, respectively) (Tables 4 and 30-minute reallocation figures - Additional File 2). Similar associations were observed for SDQ sub-scales of conduct problems, hyperactivity and emotional problems (light-intensity activities and games only). Reallocating 10 min of sleep or quiet play to screen time was associated with worse total difficulties (Δ = 0.04; 95% CI 0.01, 0.08; Δ = 0.04; 95% CI 0.02, 0.07, respectively). Similar associations were observed with each of the SDQ sub-scales except emotional problems. Emotional problems scores were worse when 10 min of energetic play was replaced with quiet play (Δ = 0.07; 95% CI 0.01, 0.14) or screen time (Δ = 0.08; 95% CI 0.02, 0.14). All other significant associations were the same as those described in the main results.

**Table 4:** Estimated changes (95% CI) in SDQ total difficulties and subscale scores associated with 10-minute reallocations between movement behaviours

10-minute reallocation	Total difficulties	Emotional problems	Peer problems	Conduct problems	Hyperactivity	Prosocial behaviour
QP to SLP	0.00 (-0.03, 0.03)	0.00 (-0.01, 0.01)	0.00 (-0.01, 0.00)	0.00 (-0.01, 0.02)	0.00 (-0.02, 0.01)	0.01 (0.00, 0.02)
SCR to SLP	**-0.05 (-0.08**,** -0.01)**	0.00 (-0.01, 0.01)	**-0.01 (-0.02**,** 0.00)**	-0.01 (-0.02, 0.00)	**-0.02 (-0.03**,** 0.00)**	**0.01 (0.00**,** 0.03)**
LIAG to SLP	**-0.10 (-0.14**,** -0.05)**	-0.01 (-0.03, 0.00)	**-0.02 (-0.03**,** 0.00)**	-0.02 (-0.04, 0.00)	**-0.05 (-0.07**,** -0.02)**	0.01 (-0.01, 0.03)
ENER to SLP	0.12 (-0.09, 0.32)	**0.08 (0.01**,** 0.14)**	0.04 (-0.03, 0.10)	-0.01 (-0.09, 0.07)	0.01 (-0.09, 0.12)	0.01 (-0.07, 0.10)
SLP to QP	0.00 (-0.02, 0.03)	0.00 (-0.01, 0.01)	0.00 (0.00, 0.01)	0.00 (-0.01, 0.01)	0.01 (-0.01, 0.02)	-0.01 (-0.02, 0.00)
SCR to QP	**-0.04 (-0.07**,** -0.02)**	-0.01 (-0.01, 0.00)	-0.01 (-0.02, 0.00)	**-0.01 (-0.02**,** 0.00)**	**-0.01 (-0.03**,** 0.00)**	0.01 (0.00, 0.02)
LIAG to QP	**-0.09 (-0.13**,** -0.05)**	**-0.01 (-0.03**,** 0.00)**	-0.01 (-0.02, 0.00)	**-0.02 (-0.04**,** -0.01)**	**-0.04 (-0.06**,** -0.02)**	0.00 (-0.02, 0.02)
ENER to QP	0.12 (-0.08, 0.32)	**0.07 (0.01**,** 0.14)**	0.04 (-0.02, 0.10)	-0.01 (-0.09, 0.07)	0.02 (-0.08, 0.12)	0.01 (-0.07, 0.09)
SLP to SCR	**0.04 (0.01**,** 0.08)**	0.00 (-0.01, 0.01)	**0.01 (0.00**,** 0.02)**	0.01 (0.00, 0.02)	**0.02 (0.00**,** 0.03)**	**-0.01 (-0.03**,** 0.00)**
QP to SCR	**0.04 (0.02**,** 0.07)**	0.01 (0.00, 0.01)	0.01 (0.00, 0.02)	**0.01 (0.00**,** 0.02)**	**0.01 (0.00**,** 0.03)**	-0.01 (-0.02, 0.00)
LIAG to SCR	**-0.05 (-0.10**,** -0.01)**	-0.01 (-0.02, 0.01)	0.00 (-0.02, 0.01)	-0.01 (-0.03, 0.01)	**-0.03 (-0.05**,** -0.01)**	-0.01 (-0.02, 0.01)
ENER to SCR	0.16 (-0.04, 0.36)	**0.08 (0.02**,** 0.14)**	0.05 (-0.02, 0.11)	0.00 (-0.08, 0.08)	0.03 (-0.07, 0.13)	0.00 (-0.08, 0.08)
SLP to LIAG	**0.09 (0.05**,** 0.14)**	0.01 (0.00, 0.03)	**0.02 (0.00**,** 0.03)**	0.02 (0.00, 0.04)	**0.05 (0.02**,** 0.07)**	-0.01 (-0.03, 0.01)
QP to LIAG	**0.09 (0.05**,** 0.13)**	**0.01 (0.00**,** 0.03)**	0.01 (0.00, 0.02)	**0.02 (0.01**,** 0.04)**	**0.04 (0.02**,** 0.06)**	0.00 (-0.02, 0.02)
SCR to LIAG	**0.05 (0.00**,** 0.09)**	0.01 (-0.01, 0.02)	0.00 (-0.01, 0.02)	0.01 (-0.01, 0.03)	**0.03 (0.01**,** 0.05)**	0.01 (-0.01, 0.03)
ENER to LIAG	0.21 (-0.01, 0.43)	**0.09 (0.02**,** 0.16)**	0.05 (-0.02, 0.12)	0.01 (-0.08, 0.10)	0.06 (-0.05, 0.17)	0.01 (-0.08, 0.10)
SLP to ENER	-0.09 (-0.25, 0.08)	**-0.06 (-0.11**,** -0.01)**	-0.03 (-0.08, 0.02)	0.01 (-0.06, 0.07)	-0.01 (-0.09, 0.07)	-0.01 (-0.08, 0.05)
QP to ENER	-0.09 (-0.25, 0.07)	**-0.06 (-0.11**,** -0.01)**	-0.03 (-0.08, 0.02)	0.01 (-0.05, 0.08)	-0.01 (-0.09, 0.07)	0.00 (-0.07, 0.06)
SCR to ENER	-0.13 (-0.29, 0.03)	**-0.06 (-0.12**,** -0.01)**	-0.04 (-0.09, 0.01)	0.00 (-0.07, 0.06)	-0.03 (-0.11, 0.05)	0.00 (-0.06, 0.07)
LIAG to ENER	**-0.18 (-0.36**,** 0.00)**	**-0.07 (-0.13**,** -0.02)**	-0.04 (-0.10, 0.01)	-0.01 (-0.08, 0.06)	-0.06 (-0.15, 0.03)	-0.01 (-0.08, 0.07)

Significant associations (*p* < 0.05) are bolded

Models adjusted for child age and sex and parent education

Lower scores are better for all scores except prosocial behaviour where a higher score is better

*SLP *Sleep, *SCR *screen-based sedentary behaviour, *QP *quiet play-based sedentary behaviour, *LIAG *light-intensity activities and games, *ENER *energetic play

## Discussion

This study provides new insights into the relationships between preschool children’s 24-hour movement behaviour compositions and their social-emotional development. Overall, more time spent in sleep and quiet play relative to other movement behaviours was associated with lower total difficulties, hyperactivity, conduct problems (quiet play only) and peer problems (sleep only) scores. Moreover, spending more time in energetic play relative to other movement behaviours was associated with fewer emotional problems. In contrast, spending more time in light-intensity activities and games and sedentary relative to other movement behaviours was associated with worse SDQ scores. No associations were found between daily screen time (relative to other movement behaviours) and SDQ scores.

Our findings partially align with the three studies to date examining preschooler movement behaviour compositions and social-emotional development. A small study of 3–5-year-olds (*n* = 95) found moderate-to-vigorous physical activity, relative to other movement behaviours was positively associated with sociability and negatively associated with internalising problems [20]. In contrast Brown and colleagues observed a negative relationship between sedentary time (relative to light and moderate-to-vigorous physical activity) and externalising problems [19]. Similarly, a study of 2-4-year-olds movement behaviour compositions while attending childcare showed more time spent sedentary compared with other movement behaviours was related to better psychosocial function [21]. Methodological differences (e.g., social-emotional development measures, processing of device-based data) and limitations (e.g., small samples sizes) between these studies and the current study likely explain variation in the findings. Further studies using large representative samples of 24-hour device-based measures of movement behaviours with validated machine-learning classification models are required.

Our findings also showed that a 10-minute/day reallocation away from sedentary behaviour and light-intensity activities and games towards sleep resulted in better SDQ scores. Furthermore, reallocating 10-minutes/day to energetic play from any of the other movement behaviours was associated with lower emotional problems scores. In support of our findings, Kuzik and colleagues found reallocating time from any other movement behaviour to moderate-to-vigorous physical activity was positively associated with sociability and cognitive self-regulation in young children [20]. In contrast, other studies have found no significant substitution effects between movement behaviour compositions and preschooler social-emotional development [19, 21]. Overall, our findings suggest reallocating some daily time spent sedentary and in light-intensity activities and games to sleep and energetic play could be beneficial for young children’s social-emotional development.

The current study also sought to understand the impact of different types of sedentary behaviours (i.e., screen time compared with quiet play) on the relationship between movement behaviour composition and preschooler social-emotional development. A 10-minute/day reallocation away from screen time and light-intensity activities and games to quiet play resulted in better SDQ scores. However, a 10-minute/day reallocation of energetic play to quiet play was associated with worse emotional problems scores. In addition, the reallocation of sleep and energetic play to screen time was associated with worse SDQ scores. These findings highlight the importance of differentiating between types (quality) of sedentary behaviours when examining the relationships between movement behaviour compositions and child outcomes. They also suggest reallocating some daily time spent in light-intensity activities and games and screen time (but not energetic play) to quiet play could be beneficial for preschooler social-emotional development. Future research should move beyond the general category of sedentary time to examine the specific impacts of different types such as screen time (e.g., sedentary, active, education-based screen time) and quiet play.

The practical significance of these findings require consideration. For example, a 100-minute reallocation away from light-intensity activities and games to sleep would result in a 1-unit decrease in the total difficulties score which has clinical significance [25] However, given the sample’s mean daily sleep of 11.5 h is in line with the preschooler sleep guidelines of 10–13 h/day [42], an additional 100-minute reallocation would result in mean daily sleep being higher than the sleep guidelines and thus may not be practical. Furthermore, a 100-minute reallocation would reduce mean daily light-intensity activities and games time from 6.3 to 4.6 h/day. This represents a significant reduction in daily time spent in light-intensity active play which could have negative consequences for other aspects of a child’s development. Future CoDA research should explore the concept of the ‘Goldilocks Day’, which aims to identify an optimal distribution of 24-hour time use across different health and development domains [43]. 

Overall, this study provides a novel way of understanding how preschool children’s movement behaviours affect their social-emotional development. By integrating multiple outcome measures researchers can better identify the ideal patterns of daily time use that support developmental goals. Such findings could inform national (and international) 24-hour Movement Guidelines for the Early Years as well as early years interventions to improve child development and later life outcomes [44]. Moreover, future movement behaviour composition focused interventions should consider a systems thinking approach to support intervention implementation, effectiveness and sustainability.

### Strengths and limitations

Key strengths include the large sample size and use of comprehensive device-based measures of preschooler movement behaviours. The use of random forest physical activity classification models to process device-based movement behaviour data enabled a more versatile and accurate capture of preschooler movement behaviour types. The use of complementary parent-report data to differentiate types of sedentary behaviour was also a strength. However, the parent-report data was a proxy measure for screen/non-screen sedentary time and did not capture sedentary time that is not leisure-based (e.g., sitting to eat, transport). Detailed weekly time-use data are needed to fully account for all types of sedentary behaviours and their contributions to 24-hour movement behaviour compositions. A further potential limitation relates to the temporality of when the data was collected, given the impact significant technological advances have had on children’s screen use.

## Conclusions

This study emphasises the importance of considering 24-hour movement behaviour compositions for preschooler’s social-emotional development. Prioritising sleep, incorporating more energetic play, and promoting quiet play while minimising screen time are important strategies for promoting social-emotional development in this young age group. This study also highlights complexities in reallocating movement behaviours as it relates to clinically significant improvements in preschooler’s social-emotional development compared with meeting 24-hour movement behaviour guidelines, underscoring the need for systems based approaches to changing movement behaviour compositions and subsequent health and development outcomes. Future research should also adopt longitudinal designs with a more comprehensive assessment of diverse movement behaviour compositions, including different types of sedentary behaviour, to strengthen causal inferences and inform evidence-based interventions for promoting health and development in young children.

## Supplementary Information

Additional file 1.

Additional file 2.

## Data Availability

The de-identified data generated from this study will be available for analytic purposes by researchers and students if approved by the Principal Investigator and relevant ethics committees, from 12 months following the anticipated project end date.

## Abbreviations

PLAYCE: PLAY Spaces and Environments for Children’s Physical Activity
CoDA: Compositional Data Analysis
ECEC: Early Childhood Education and Care
SDQ: Strengths and Difficulties Questionnaire
Ilr: Isometric Log-Ratios
IQR: Interquartile Range
SD: Standard Deviation
CI: Confidence Interval
