# Supplementary material for: Compositional movement behaviours and preschool children’s social-emotional development
Source: Int J Behav Nutr Phys Act. 2026 Apr 16;23:54. doi: 10.1186/s12966-026-01911-2 (PMC13224659; doi:10.1186/s12966-026-01911-2)
Supplement: Supplementary file 1 — Additional file 1. [file 12966_2026_1911_MOESM1_ESM.docx]

**Additional File 1: One-to-one reallocations for SDQ subscales**


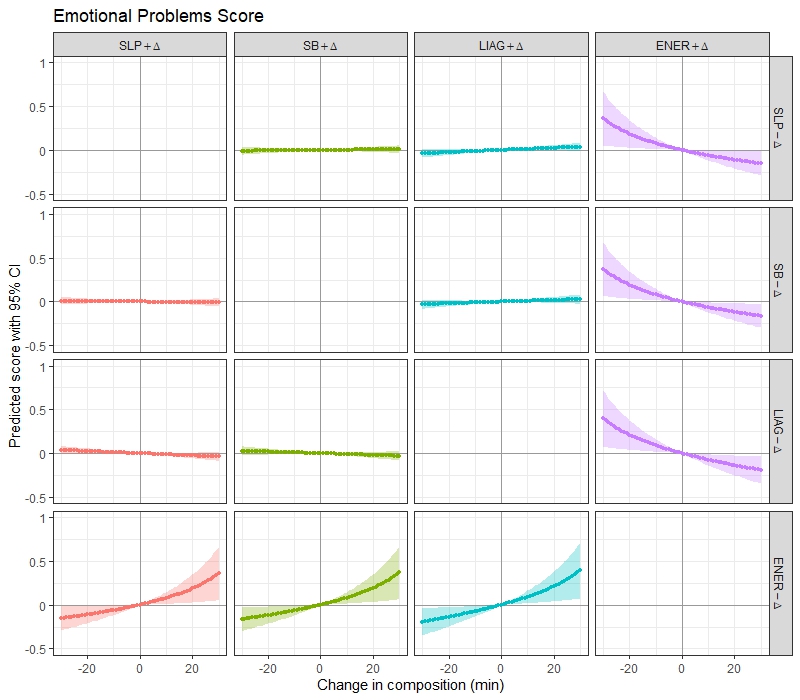


Figure 1. Effects of one-to-one reallocations of time between movement behaviours on SDQ Emotional Problems score; Δ = change.

SLP=Sleep, SB=Sedentary behaviour, LIAG=light-intensity activities and games, ENER=energetic play.


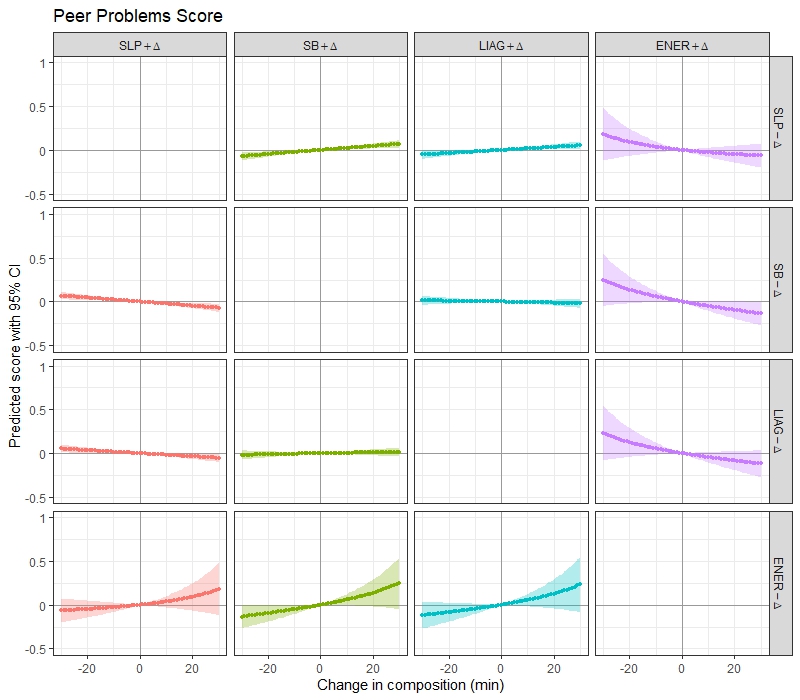


Figure 2. Effects of one-to-one reallocations of time between movement behaviours on SDQ Peer Problems score; Δ = change.

SLP=Sleep, SB=Sedentary behaviour, LIAG=light-intensity activities and games, ENER=energetic play.


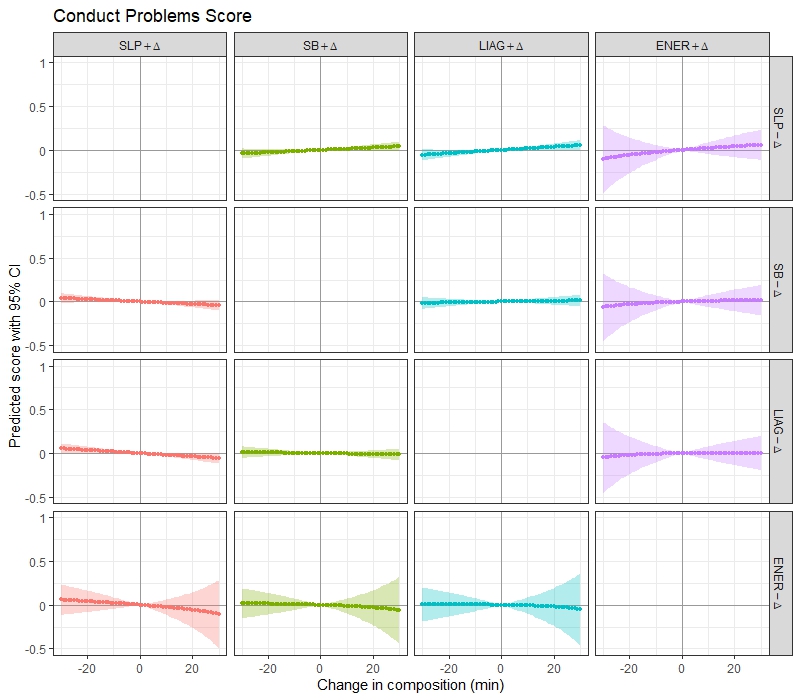


Figure 3. Effects of one-to-one reallocations of time between movement behaviours on SDQ Conduct Problems score; Δ = change.

SLP=Sleep, SB=Sedentary behaviour, LIAG=light-intensity activities and games, ENER=energetic play.


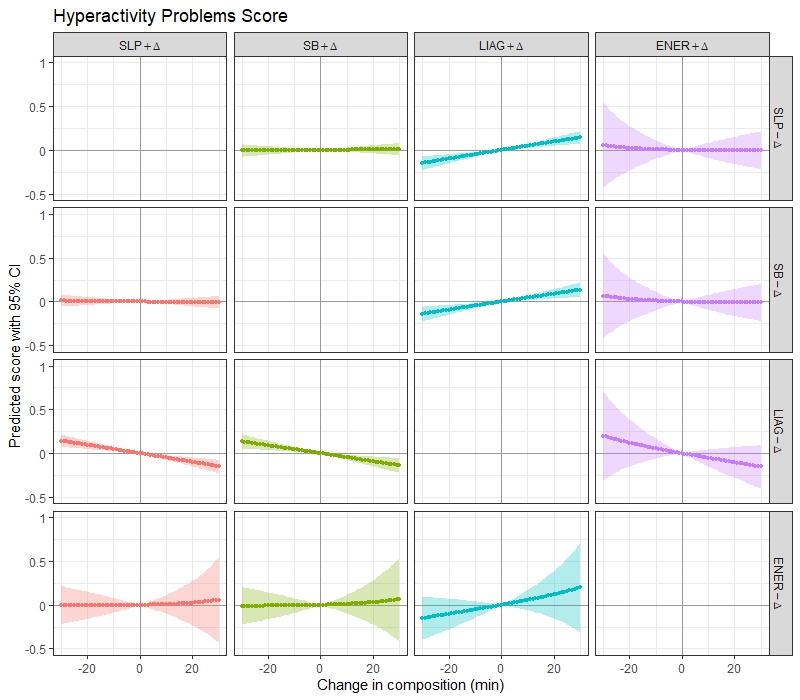


Figure 4. Effects of one-to-one reallocations of time between movement behaviours on SDQ Hyperactivity Problems score; Δ = change.

SLP=Sleep, SB=Sedentary behaviour, LIAG=light-intensity activities and games, ENER=energetic play.


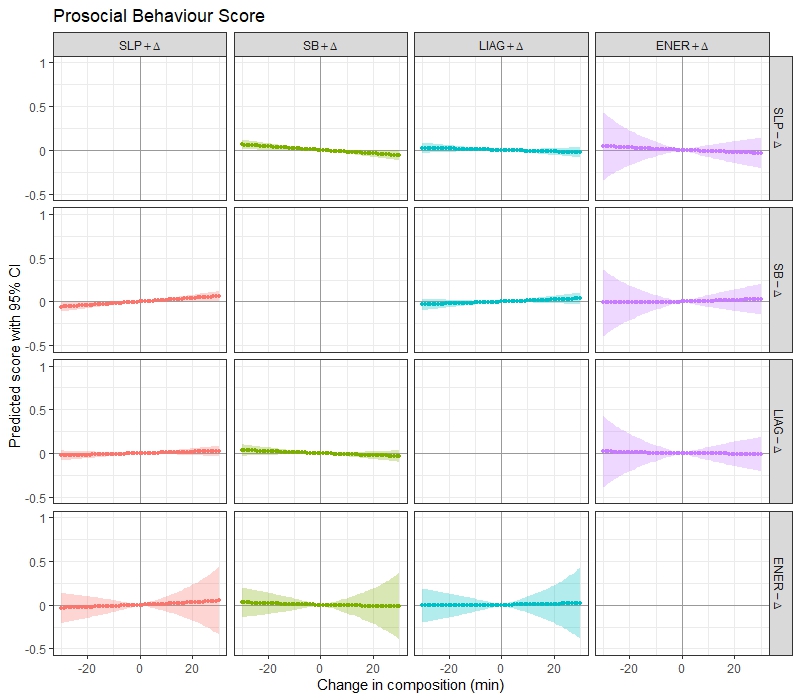


Figure 5. Effects of one-to-one reallocations of time between movement behaviours on SDQ Prosocial Behaviours score; Δ = change.

SLP=Sleep, SB=Sedentary behaviour, LIAG=light-intensity activities and games, ENER=energetic play.
