# Supplementary material for: Compositional movement behaviours and preschool children’s social-emotional development
Source: Int J Behav Nutr Phys Act. 2026 Apr 16;23:54. doi: 10.1186/s12966-026-01911-2 (PMC13224659; doi:10.1186/s12966-026-01911-2)
Supplement: Supplementary file 2 — Additional file 2. [file 12966_2026_1911_MOESM2_ESM.docx]

**Additional File 2: Seconday Analysis: One-to-one reallocations for SDQ total difficulties and sub-scale scores, after disaggregating sedentary time into screen time and quiet play
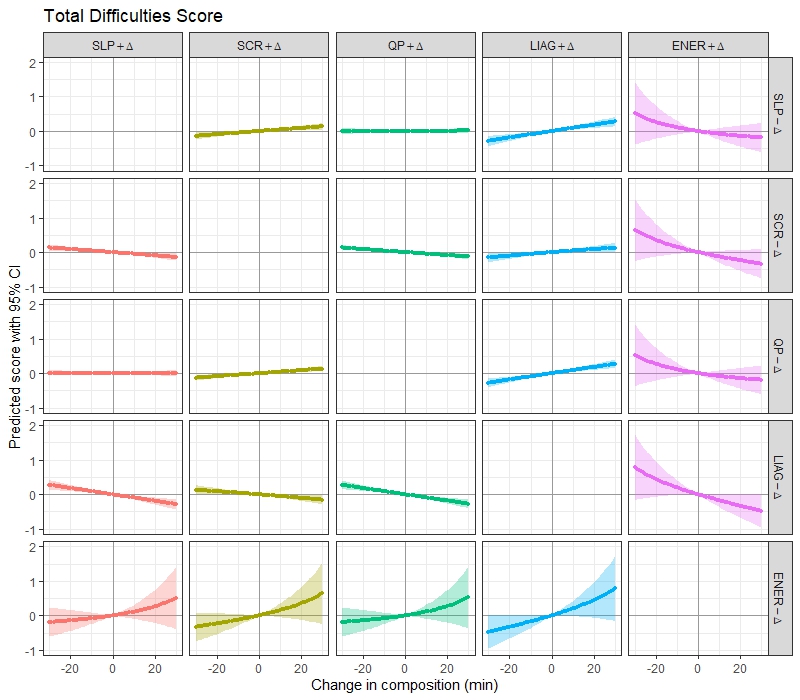
**

Figure 6. Effects of one-to-one reallocations of time between movement behaviours on SDQ Total Difficulties score after disaggregating sedentary time to screen time and quiet play; Δ = change.

SLP=Sleep, SCR=screen-based sedentary behaviour, QP=quiet play-based sedentary behaviour, LIAG=light-intensity activities and games, ENER=energetic play.


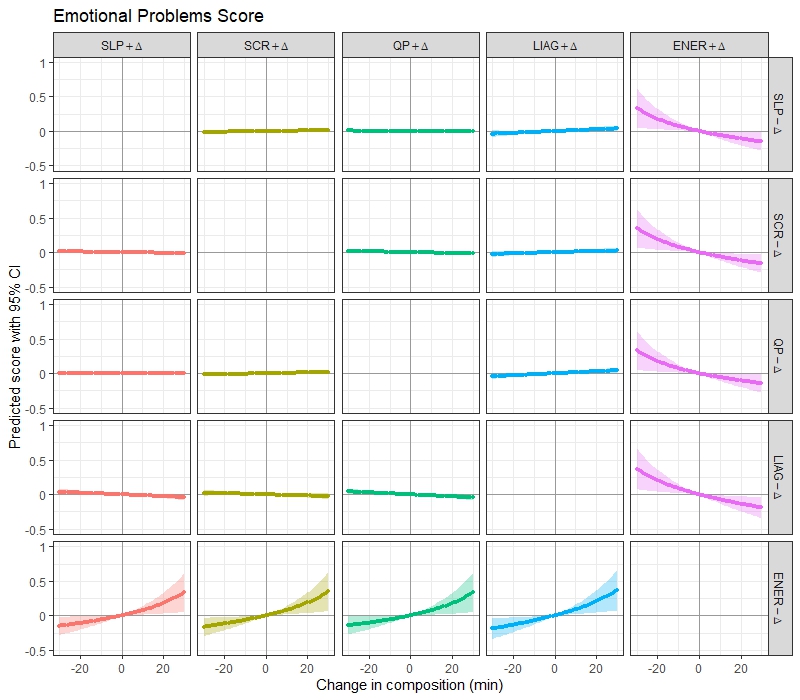


Figure 7. Effects of one-to-one reallocations of time between movement behaviours on SDQ Emotional Problems score after disaggregating sedentary time to screen time and quiet play; Δ = change.

SLP=Sleep, SCR=screen-based sedentary behaviour, QP=quiet play-based sedentary behaviour, LIAG=light-intensity activities and games, ENER=energetic play.


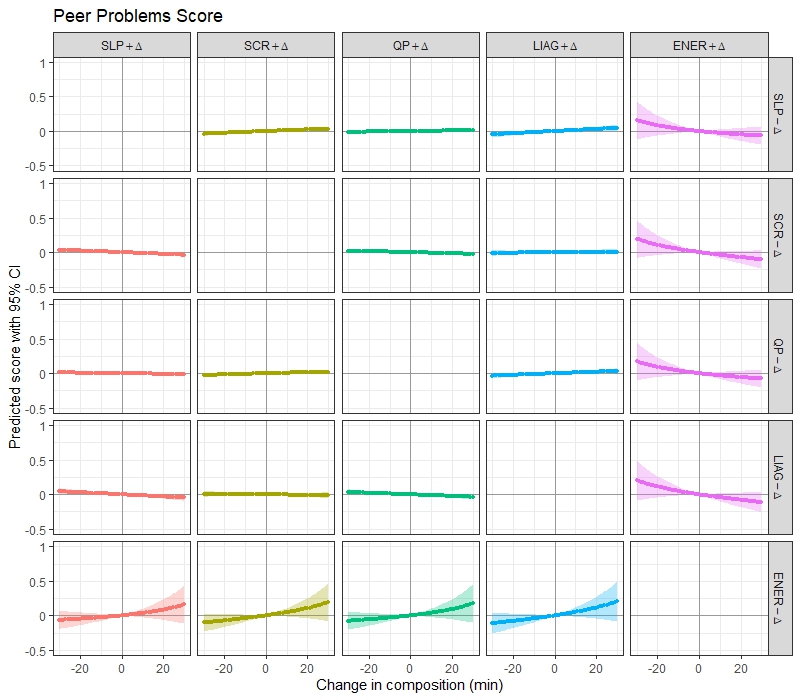


Figure 8. Effects of one-to-one reallocations of time between movement behaviours on SDQ Peer Problems score after disaggregating sedentary time to screen time and quiet play; Δ = change.

SLP=Sleep, SCR=screen-based sedentary behaviour, QP=quiet play-based sedentary behaviour, LIAG=light-intensity activities and games, ENER=energetic play.


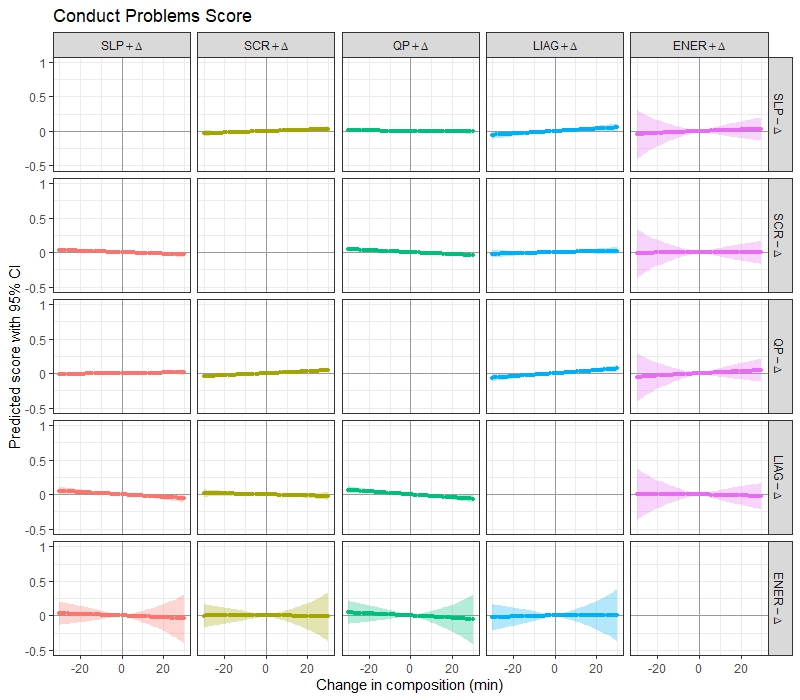


Figure 9. Effects of one-to-one reallocations of time between movement behaviours on SDQ Conduct Problems score after disaggregating sedentary time to screen time and quiet play; Δ = change.

SLP=Sleep, SCR=screen-based sedentary behaviour, QP=quiet play-based sedentary behaviour, LIAG=light-intensity activities and games, ENER=energetic play.


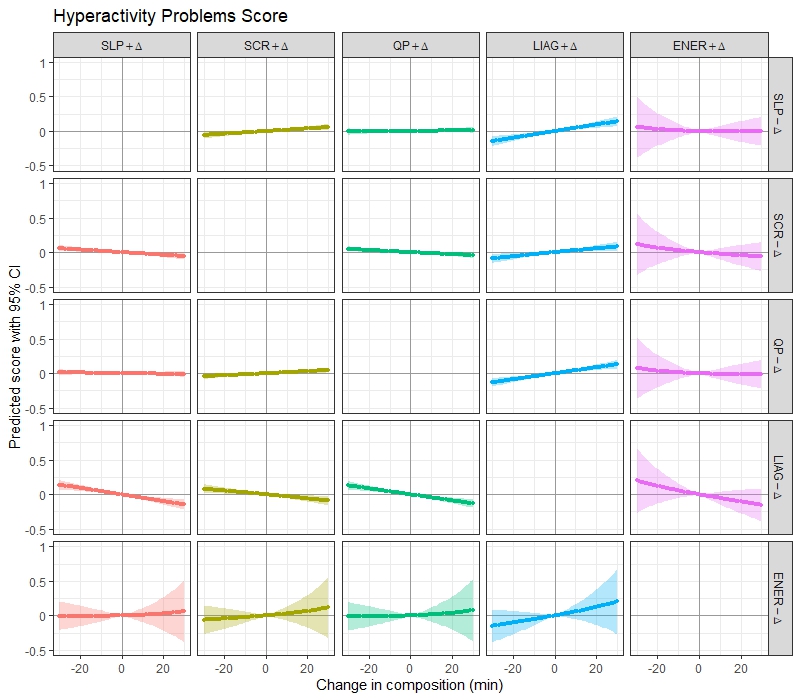


Figure 10. Effects of one-to-one reallocations of time between movement behaviours on SDQ Hyperactivity Problems score after disaggregating sedentary time to screen time and quiet play; Δ = change.

SLP=Sleep, SCR=screen-based sedentary behaviour, QP=quiet play-based sedentary behaviour, LIAG=light-intensity activities and games, ENER=energetic play.


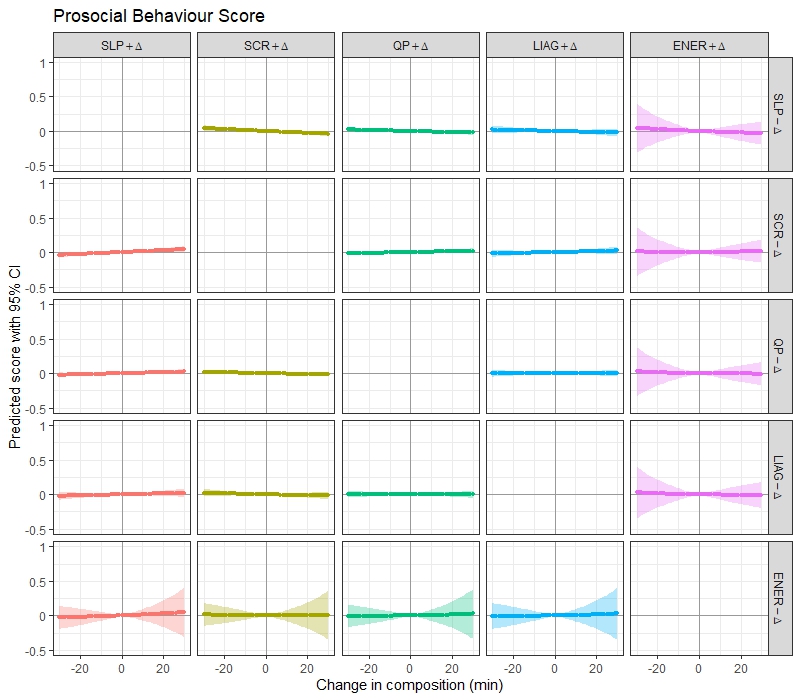


Figure 11. Effects of one-to-one reallocations of time between movement behaviours on SDQ Prosocial Behaviour score after disaggregating sedentary time to screen time and quiet play; Δ = change.

SLP=Sleep, SCR=screen-based sedentary behaviour, QP=quiet play-based sedentary behaviour, LIAG=light-intensity activities and games, ENER=energetic play.
